# Anti-Immunoglobulin E Therapy

**DOI:** 10.1097/WOX.0b013e318187a310

**Published:** 2008-10-15

**Authors:** Manav Segal, Jeffrey R Stokes, Thomas B Casale

**Affiliations:** 1 Division of Allergy and Immunology, Department of Medicine, Creighton University School of Medicine, 601 North 30th Street, Suite 3M100, Omaha, NE 68131

**Keywords:** anti-IgE, omalizumab, asthma, safety

## Abstract

The importance of immunoglobulin E (IgE) in atopic disorders such as asthma, allergic rhinitis, food allergies, and atopic dermatitis is well established. Elevation of total serum IgE is typically found in many atopic patients, and in predisposed individuals, allergen-specific IgE is produced. The availability of humanized monoclonal antibodies against IgE has provided a new therapeutic option and tool to explore the role IgE in allergic diseases and the effects of inhibiting IgE itself. Omalizumab is a humanized, monoclonal antibody that recognizes and binds to the Fc portion of the IgE molecule. Administration of omalizumab results in a rapid and substantial decrease in free IgE in serum. Consequently, the activity of cell populations involved in allergic inflammation, including mast cells, eosinophils, basophils, and antigen-presenting cells, is affected as well. Clinically, anti-IgE therapy has already been proven to be useful in the treatment of asthma and allergic rhinitis. The aim of this review is to provide an overview of the mechanisms of action of anti-IgE therapy as well as its efficacy in the treatment of allergic diseases, especially asthma. Considerations regarding dosing and safety of omalizumab will be addressed as well.

## Background

**Figure 1:**
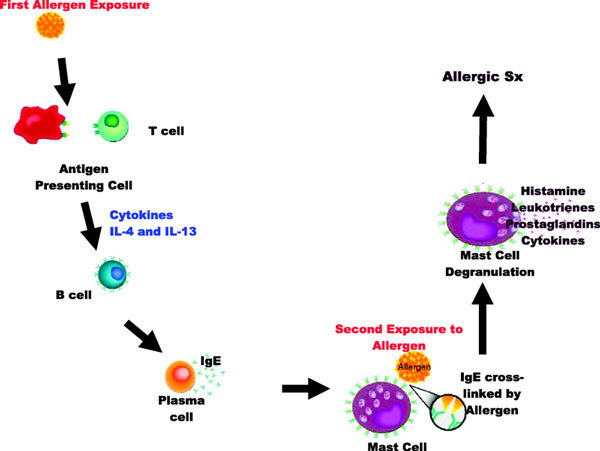
**Immunoglobulin E and the allergic cascade (IL indicates interleukin; Sxs, symptoms)**. After allergen sensitization, cytokines stimulate B cells to produce allergen-specific IgE. Immunoglobulin E molecules then circulate and bind to high-affinity IgE receptors (Fc*ε*RI) on the surface of mast cells and basophils. Activation of mast cells and basophils by cross-linking of the surface IgE molecules leads to degranulation and release of mediators. Adapted from Brownell and Casale [2].

Immunoglobulin E (IgE), originally described in 1967 by Ishizaka et al,[1] is well established to be important in atopic disorders such as asthma, allergic rhinitis, food allergies, and atopic dermatitis. Immunoglobulin E binds to the high-affinity IgE receptor, Fc*ε*RI, and is subsequently expressed on the surface of a number of key inflammatory cells, including mast cells, basophils, and dendritic cells. When allergen binds to the Fab portion of the IgE molecule, cross-linking of 2 adjacent IgE molecules on the surface of allergic effector cells (in particular mast cells and basophils) initiates intracellular signaling pathways that result in the release of preformed and newly synthesized mediators. This type 1 hypersensitivity reaction is central to the pathogenesis of atopic disorders [2] (Figure 1).

Thus far, chronic therapy for allergic diseases has largely been limited to blocking the effects of specific mediators (eg, leukotriene modifiers and anti-histamines) or the use of corticosteroids to reduce the consequences of mediator release on the inflammatory cascade. More recently, however, the availability of humanized monoclonal antibodies against IgE has provided a new therapeutic option and tool to more closely explore the role of IgE in allergic diseases and the effects of inhibiting IgE itself.

**Figure 2:**
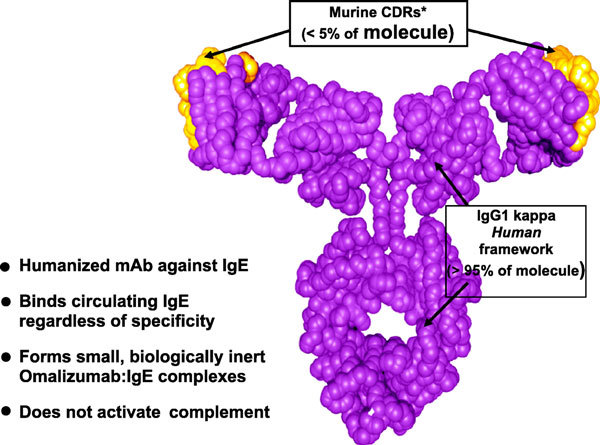
**Omalizumab structure**. Omalizumab is a recombinyant humanized monoclonal antibody composed of 95% human sequence and 5% murine sequence. *CDR indicates complementarity-determining region. Adapted from Boushey [3].

Omalizumab is a humanized, monoclonal antibody that recognizes and binds to IgE. Approximately 5% of omalizumab is composed of murine sequences (the antigen recognition portion of the molecule) that were engrafted onto a human IgG1κ framework (Figure 2) [3-5]. Omalizumab binds to the CH3 domain of the IgE molecule, which is conserved among all IgE molecules [6]. This is the same site by which IgE binds to Fc*ε*RI. Because omalizumab binds to the same site that IgE molecules use to attach to Fc*ε*RI, it cannot cross-link cell surface-expressed IgE. Thus, omalizumab can bind only to soluble IgE and therefore cannot precipitate degranulation of effector cells via this mechanism [7].

### IgE and IgE receptors

Immunoglobulin E is present in the serum in far lesser amounts than IgG, IgM, or IgA. The half-life of IgE in the serum is only 2 days. The expression of Fc*ε*RI on the surface of critical effector cells, such as basophils, is up-regulated by IgE. This effect likely occurs through the direct interaction of IgE with Fc*ε*RIα [8]. Another IgE receptor, Fc*ε*RII (CD23), whose role is less certain, binds with much lower affinity to IgE. Fc*ε*RII seems to have opposing effects dependent on whether the molecule is expressed on the cell surface or exists free in the serum. Soluble Fc*ε*RII up-regulates the production of IgE through interaction with CD21 in the B-cell coreceptor. In contrast, ligation of cell surface-expressed Fc*ε*RII by IgE seems to inhibit IgE production [9].

## Mechanism of action

Administration of omalizumab results in a rapid and substantial decrease in free IgE in serum [10]. By virtue of this dramatic reduction in serum-free IgE levels, omalizumab decreases the expression of Fc*ε*RI on several cell types [10-13]. A 99% reduction in free serum IgE levels has been noted within 2 hours of omalizumab administration. Furthermore, within 3 months of therapy, human basophil responsiveness (histamine releasability) was reduced by 90% [11]. Omalizumab administration results in reductions in allergen-induced nasal challenge responses and expression of Fc*ε*RI on basophils within 7 days [11].

Antigen presentation by dendritic cells is facilitated by the surface expression of Fc*ε*RI [14]. Two subtypes of dendritic cells, DC1 and DC2, seem to be instrumental in the phenotypic development of Th1 and Th2 cells, respectively [15]. The expression of Fc*ε*RI on dendritic cells is greater in patients with asthma than nonatopic controls and correlates with serum IgE levels [16]. In ragweed-sensitive patients with seasonal allergic rhinitis treated with omalizumab, both DC1 and DC2 cells showed a significant decrease in Fc*ε*RI expression as early as day 7 that persisted through day 42 of treatment. The decrease in Fc*ε*RI expression also correlated with a decrease in serum-free IgE as well as basophil Fc*ε*RI expression [12]. Recent data showed that omalizumab reduces Fc*ε*R1 expression on monocytes as well [17]. These results suggest that omalizumab may have a significant effect on the sensitization phase of the allergic response by regulation of Fc*ε*RI expression on dendritic cells and monocytes.

Unlike the rapid decrease in Fc*ε*RI expression induced on basophils and dendritic cells, omalizumab's effect on cutaneous mast cell Fc*ε*RI expression seems to occur more gradually. A small study evaluated the effect of omalizumab on intradermal allergen skin test titration and on Fc*ε*RI expression in skin biopsy samples. Omalizumab had no effect on Fc*ε*RI on day 7; however, by day 70, there was a 90% reduction in Fc*ε*RI expression in skin biopsy specimens. There was no change in the number of tryptase-positive cells in the biopsy specimens. The authors interpreted this as an omalizumab-induced decrease in the expression of Fc*ε*RI by cutaneous mast cells [13]. In general, omalizumab has a weak and delayed effect on allergen-induced immediate skin test responses. This has resulted in the unreliability of skin test responses as a surrogate biomarker to regulate and explore dosing effects of this agent. However, the inhibitory effects of omalizumab on late-phase skin responses are more rapid and profound [13,18].

### Omalizumab's effects on markers of airway inflammation

Nitric oxide (NO) has been identified as an important noninvasive marker of airway inflammation in patients with asthma. The effects of omalizumab on exhaled NO have been examined in 29 children with moderate-to-severe allergic asthma. Subjects randomized to omalizumab showed a significant reduction in exhaled NO from baseline to the end of the 52-week study period (*P *= 0.032) despite a substantial reduction in the dose of inhaled corticosteroids [19].

Omalizumab treatment reduces blood eosinophil levels in patients with seasonal allergic rhinitis correlating with reduced levels of serum-free IgE [20]. Similar decreases in both blood and sputum eosinophils compared with baseline values have been seen in patients with allergic asthma during omalizumab therapy [21,22]. Omalizumab treatment has also been shown to decrease B-lymphocyte counts under experimental conditions. No significant differences were noted in the other lymphocyte subpopulations [23].

Omalizumab's effect on inflammatory cells in bronchial biopsies as well as on sputum eosinophils has been evaluated. Forty-five patients with mild-to-moderate-persistent asthma with sputum eosinophilia more than 2% were treated with omalizumab (n = 22) or placebo (n = 23) for 16 weeks. Subjects underwent sputum induction and bronchoscopy with bronchial biopsy before and after treatment. Treatment with omalizumab resulted in a significant decrease in the mean percentage of sputum eosinophils from 6.6% to 1.7% (*P *= 0.05 vs placebo). This was associated with a significant reduction in tissue eosinophils; cells positive for Fc*ε*R1; CD3^+^, CD4^+^, and CD8^+ ^T lymphocytes; B lymphocytes; and cells staining positive for interleukin 4 (IL-4); but not improvement in airway hyperresponsiveness to methacholine [24]. The dichotomy between omalizumab's effects on airway inflammation versus hyperresponsiveness suggested to the authors that IgE and/or eosinophils may not be causally linked to airway hyperresponsiveness to methacholine in mild-to-moderate asthma. Omalizumab has shown inconsistent effects on airway hyperresponsiveness [24-26]. However, consistent with the lung anti-inflammatory effects of omalizumab, early proof of concept studies documented the ability of omalizumab to inhibit both early and late allergen-induced airway responses and the consequent increase in sputum eosinophils [21,26].

Thirty-five patients with moderate-to-severe allergic asthma were treated with omalizumab in addition to baseline ICS, and circulating cytokines were measured. Levels of IL-13 were significantly decreased in omalizumab compared with placebo-treated patients, whereas IL-5 and IL-8 had nonsignificant decreases. Levels of IL-6, IL-10, and s-ICAM were unchanged with omalizumab therapy [22].

Another possible mechanism of action involves the potential of omalizumab to promote mast cell apoptosis. The binding of different IgE molecules to Fc*ε*RI induces a spectrum of activation events in the absence of antigen. Highly cytokinergic IgEs induced production of cytokines and rendered mast cells resistant to apoptosis in an autocrine fashion [27]. Thus, by decreasing IgE and Fc*ε*R1 expression, omalizumab might lead to mast cell apoptosis, but this has not yet been demonstrated.

**Figure 3:**
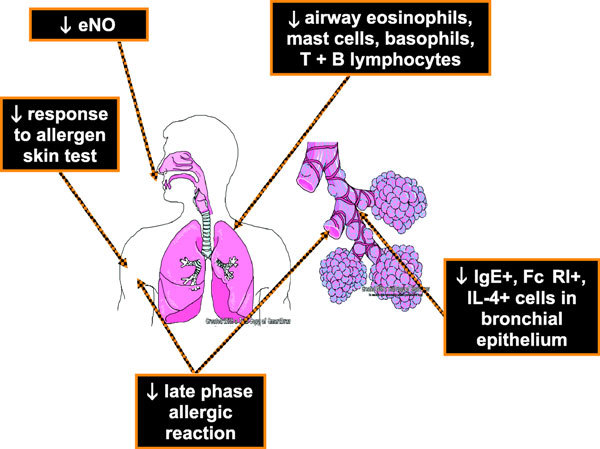
**Omalizumab mechanisms of action**.

**Figure 4:**
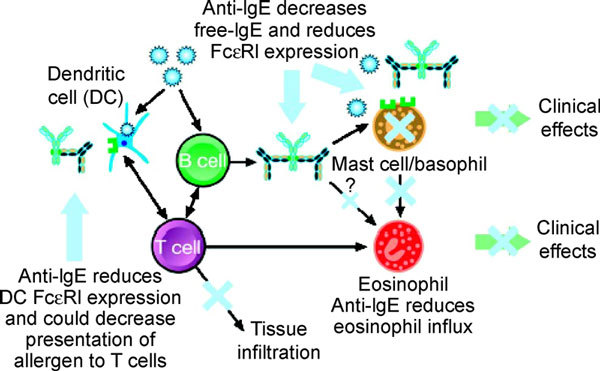
**Omalizumab mechanisms of action**. Omalizumab/anti-IgE decreases free IgE, IgE bound to Fc*ε*RI, and Fc*ε*RI expression on mast cells, basophils, dendritic cells, and monocytes. It also reduces tissue infiltration by T and B lymphocytes, eosinophils, mast cells, and basophils.

Omalizumab treatment has been shown to induce human eosinophil apoptosis in patients with allergic asthma. After 12 weeks of therapy with omalizumab, markers of eosinophil apoptosis (annexin V) were significantly increased. In addition, fewer GM-CSF^+^, IL-2^+^, and IL-13^+ ^lymphocytes were evident in omalizumab versus placebo-treated allergic asthma patients [28]. Figures 3 and 4 illustrate the mechanisms of action summarized in Table 1[29].

**Table 1:** Omalizumab: Summary of Effects on Airway Inflammation

Decreases free serum IgE
Decreases expression of Fc*ε*RI (on mast cells, basophils, dendritic cells, and monocytes)
Decreases eosinophils (serum, sputum, bronchial biopsies)
Decreases B lymphocytes
Decreases antigen-induced mediator release from basophils and mast cells
Decreases circulating IL-13
Decreases airway inflammation
Decreases FeNO

Adapted from Stokes and Casale [29].

## Omalizumab disease-specific effects

### Asthma

A number of studies have established the efficacy and safety of omalizumab for the treatment of patients with moderate-to-severe asthma leading to the US Food and Drug Administration's (FDA) approval of omalizumab for the treatment of moderate-to-severe persistent allergic asthma in patients 12 years or older.

Three phase 3 trials were conducted on a total of 1405 patients with moderate-to-severe allergic asthma [30-32]. Two trials were conducted in subjects 12 years and older treated with inhaled corticosteroids [30,32]. The third trial was conducted in children aged 6 to 12 years old receiving inhaled corticosteroids [31]. During the initial 16 weeks, omalizumab was added on to inhaled corticosteroids at a stable dose. Subjects then underwent a steroid-reduction phase lasting 12 weeks. Doses of corticosteroids were reduced by up to 25% every 2 weeks to the lowest level required for optimal disease control.

A reduction in asthma exacerbations when compared with placebo was shown in all 3 studies. Furthermore, omalizumab demonstrated a corticosteroid-sparing effect in all 3 studies. In addition, fewer asthma symptoms, less rescue medication usage, and improved quality-of-life scores were noted in the omalizumab-treated patients. In the adolescent and adult studies, omalizumab resulted in small but statistically significant improvements in peak expiratory flow and forced expiratory volume in 1 second (FEV_1_) [32].

The data from these 3 studies were later pooled to determine the effect of omalizumab on serious asthma exacerbations [33]. Significantly fewer unscheduled outpatient visits and emergency-room visits were observed in the omalizumab-treated patients compared with placebo. Hospitalizations were also significantly reduced from 3.42 events per 100 patient-years on placebo to 0.26 on omalizumab treatment.

To examine baseline characteristics predictive of a response to omalizumab, data from 1070 adolescents and adults from 2 of these phase 3 trials were pooled [34]. Patients with features suggestive of greater disease severity seemed to obtain the greatest benefit from the addition of omalizumab to their therapeutic regimen. These included patients with a history of emergency treatment of asthma in the last year, patients on 800 μg or greater inhaled beclomethasone per day, and patients with an FEV_1 _65% or less of predicted. The greatest benefits were noted in patients who had 2 or more of these characteristics.

Of 1412 patients in phase 3 trials, 254 patients were identified as high risk (ever required intubation, visited an emergency room, required overnight hospitalization, or received treatment in an ICU during the last year). Most the high-risk patients had undergone an emergency room admission with only a small proportion requiring ICU care and/or intubation. In these high-risk patients on the steroid-stable phase of the studies, those treated with omalizumab had a reduction in significant asthma exacerbation episodes by 56%. In addition, these high-risk patients treated with omalizumab were less likely to be rehospitalized due to asthma while demonstrating improvements in peak flows and asthma symptoms. Omalizumab prevented exacerbations in approximately 17 additional patients for every 100 treated. Fifty percent of potential exacerbations were prevented by treatment with omalizumab, and 5.7 patients were needed to be treated with omalizumab to maintain 1 patient free of an exacerbation [35].

Patients with severe allergic asthma who required high-dose fluticasone (≥ 1000 μg/d) with or without oral corticosteroids were treated with omalizumab. At 32 weeks, a significant reduction in the dose of inhaled corticosteroids was noted in the omalizumab group when compared with placebo (mean, 57.2% vs 43.3%; *P *= 0.003). Improved symptoms, less rescue medication usage, and improved quality of life were also observed in the omalizumab group when compared with placebo [36]. However, no significant reduction in exacerbations was observed in this study.

In practice, omalizumab is typically added to the therapeutic regimen in patients who remain poorly controlled despite maximal medical therapy. It is these more severe patients with asthma that are at greatest risk of serious complications and mortality [37]. The addition of omalizumab to maximal conventional asthma therapy was evaluated in a 52-week trial. The study involved 312 symptomatic patients with moderate-to-severe allergic asthma who were symptomatic despite treatment with high doses of inhaled corticosteroids plus long-acting β_2_-agonists, anti-leukotrienes, or oral steroids [38]. Compared with placebo, the omalizumab group showed a greater reduction of asthma exacerbations (60%), unscheduled physician visits, and days missed from work or school. A similar study evaluated 419 patients with severe uncontrolled asthma despite high dose of inhaled corticosteroids and long acting bronchodilators. Patients were randomized to receive omalizumab or placebo for 28 weeks in a double-blind, parallel-group fashion [39]. Omalizumab therapy significantly reduced the rate of significant exacerbations while decreasing emergency visits by 44%. In addition, quality-of-life scores, pulmonary functions, and asthma symptom scores improved with omalizumab add on therapy.

Another report evaluated the pooled data from 5 double-blind trials and 2 open-label studies for an analysis of the effect of add-on therapy with omalizumab on asthma exacerbations in severe patients with asthma [40]. A total of 4308 patients were included (2511 treated with omalizumab). Omalizumab decreased the rate of asthma exacerbations by 38% and also decreased the rate of emergency visits by 47%. Taken together, these studies suggest that omalizumab is effective as an add-on therapy in severe patients with asthma that are poorly controlled despite maximal medical therapy. A summary of the effects noted in patients with allergic asthma treated with omalizumab is shown in Table 2[29].

**Table 2:** Omalizumab: Demonstrated Clinical Effects on Asthma

Decreased inhaled corticosteroid doses
Decreased asthma symptoms
Decreased rescue medication usage
Decreased exacerbations
Decreased emergency room visits
Decreased hospitalizations
Improved pulmonary functions (small effect)
Increased quality of life

Adapted from Stokes and Casale [29].

### Allergic rhinitis

The effect of omalizumab was evaluated in patients with ragweed-sensitive allergic rhinitis [41]. Patients were randomized to receive 1 of 3 doses of omalizumab or placebo. Subjects treated with 300 mg omalizumab every 3 to 4 weeks demonstrated significantly lower nasal symptoms scores, rhinitis quality-of-life scores, and days missed from work or school when compared with placebo during the pollen season. In addition, significant correlations were observed between the dose of omalizumab, reduction in serum IgE and improvements in nasal symptoms, and rescue antihistamine use.

Another large seasonal allergic rhinitis study examined the therapeutic benefits of omalizumab in birch-pollen-sensitive rhinitics [42]. The omalizumab-treated group showed significant improvements over placebo in average daily symptom scores, usage of rescue antihistamines, and quality-of-life measures during birch season. A significant decrease in basal tumor necrosis factor α and albumin in the nasal lavage fluid (a marker of vascular permeability) was also shown in omalizumab-treated seasonal allergic rhinitis patients [43].

Similar clinical efficacy was demonstrated by a double-blind, randomized, placebo-controlled study involving nearly 100 patients treated with either omalizumab (n = 48) or placebo (n = 50) during Japanese cedar pollen season [44]. A later publication by the same authors directly compared the effect of omalizumab to suplatast tosilate, a drug that selectively inhibits the Th2 cytokine production from T cells, in controlling rhinoconjunctival symptoms associated with Japanese cedar pollen. The authors concluded that omalizumab was superior in preventing and controlling symptoms and reducing rescue medication use [45].

Omalizumab was also shown to be effective in the treatment of perennial allergic rhinitis when patients with moderate-to-severe disease were evaluated [46]. However, the magnitude of effect was small. The multiple upper airway improvements noted with omalizumab therapy are seen in Table 3[29].

**Table 3:** Omalizumab: Clinical Effects on Allergic Rhinitis

Decreased daily symptoms
Decreased rescue medication usage
Increased quality of life
Decreased nasal allergen challenge responses
Decreased missed school or work days

Adapted from Stokes and Casale [29].

Allergic rhinitis and asthma frequently coexist. Poor control of rhinitis may exert a detrimental effect on asthma [47]. Several studies have demonstrated improved asthma control when concomitant allergic rhinitis is properly treated [47-50]. The SOLAR study evaluated adolescents and adults with moderate-to-severe asthma on stable asthma and rhinitis therapy who also had persistent allergic rhinitis [51]. Fewer asthma exacerbations were observed in the omalizumab-treated patients than placebo-treated patients (20.6% vs 30.1%). Furthermore, more patients in the omalizumab group demonstrated a clinically significant improvement in both asthma and rhinitis quality-of-life indices (57.7% vs 40.6%). This suggests omalizumab is effective in the treatment of upper and lower airway symptoms in the same patients [51].

### Omalizumab plus immunotherapy

The rationale for using the combination of anti-IgE and allergen immunotherapy comes from preexisting data about the biologic and immunologic effects of both therapies. The immunomodulatory effects of immunotherapy are due to a number of proposed mechanisms, including blunting of seasonal increases in IgE levels, increasing allergen-specific IgG levels, shifting the balance of T-lymphocyte subsets away from a Th2 phenotype and toward a Th1 phenotype, and production of IL-10 [52-57]. However, immunotherapy is associated with the risk of allergic reactions to the extract injections.

The addition of omalizumab to standard maintenance dose immunotherapy was evaluated in 221 children and adolescents with sensitization to birch and grass allergen [58]. During birch season, both birch and grass immunotherapy plus omalizumab groups had decreased symptoms by 39% in the group treated with irrelevant immunotherapy (grass) and by 48% in the birch immunotherapy group compared with irrelevant immunotherapy and placebo. Similar results were seen in grass season with irrelevant (birch) immunotherapy and omalizumab decreasing symptoms by 45%, whereas patients on grass immunotherapy and omalizumab had a reduction in symptom scores by 71% compared with irrelevant immunotherapy and placebo. When these findings were further analyzed for the grass-pollen-allergic children, it was noted that omalizumab plus immunotherapy-treated groups had significantly diminished rescue medication usage and number of symptomatic days when compared with either omalizumab or immunotherapy alone [59]. The combined treatment with both omalizumab and grass immunotherapy was more effective than omalizumab alone.

The primary objective of a more recent study was to determine whether omalizumab, given 9 weeks before rush immunotherapy, followed by 12 weeks of dual omalizumab and immunotherapy was more effective than rush immunotherapy followed by immunotherapy alone in ragweed-allergic patients [60]. A major secondary objective was whether omalizumab improved the safety of rush immunotherapy. Omalizumab improved both the efficacy and the safety of immunotherapy. Patients receiving omalizumab plus immunotherapy had fewer adverse events than those receiving immunotherapy alone. Post hoc analysis of groups receiving immunotherapy demonstrated that the addition of omalizumab resulted in a 5-fold decrease in risk of anaphylaxis caused by RIT (odds ratio, 0.17; *P *= 0.026). On an intention-to-treat basis, patients receiving both omalizumab and immunotherapy showed a significant improvement in severity scores during the ragweed season compared with those receiving immunotherapy alone (0.69 vs 0.86; *P *= 0.044). Thus, combined therapy with omalizumab and allergen immunotherapy may be an effective strategy to permit more rapid and higher doses of allergen immunotherapy to be given more safely and with greater efficacy to patients with allergic diseases [60].

### Atopic dermatitis

Atopic dermatitis is a chronic inflammatory disease of the skin that affects 10% to 20% of children and 1% to 3% of adults [61]. In the allergic form of atopic dermatitis, serum total IgE is elevated [62]. A few small-scale reports of omalizumab therapy have demonstrated conflicting results. A small study reported on 3 patients with severe atopic dermatitis and baseline IgE levels of 1990, 2890, and 6120 IU/mL. All of the patients noted significant improvement on omalizumab (150-450 mg every 2 weeks for 12 weeks) as early as 2 weeks after therapy was initiated [63]. Another study evaluated 7 patients with asthma, allergic rhinitis, and atopic dermatitis. Their baseline IgE levels ranged from 262 to 2020 IU/mL. Eczema symptoms were scored before therapy and after 3 and 7 months of omalizumab. Six of the 7 patients had at least moderate disease before the start of therapy but only 5 completed 7 months of therapy due to problems with insurance coverage for treatment. Improvement was noted within the first 3 months of therapy. Of the 5 remaining patients at 7 months, 3 had resolution of their eczema and 2 had only mild disease [64].

Another small study of 3 adult patients with severe atopic dermatitis and concomitant asthma, allergic rhinitis, or both were treated with omalizumab 450 mg every 2 weeks [65]. After 4 months of therapy, no improvement was noted in their atopic dermatitis symptoms. The baseline values of IgE before treatment were extremely elevated (5440, 23000, and 24400 IU/mL) [65]. In a pilot investigation published recently, low-dose omalizumab (150 mg every 2 weeks) demonstrated promising results in some of the patients with generalized atopic dermatitis. Serum total IgE levels of all patients were well over 1000 IU/mL before treatment (range, 1343-39,534 IU/mL). Six patients responded with satisfying to very good clinical response based on scoring (Scoring Atopic Dermatitis [SCORAD]). On the other hand, 5 patients showed either no relevant changes or clinical deterioration at the conclusion of the study (10 cycles of treatment) [66]. On the basis of the limited data available, the role for omalizumab in the treatment of atopic dermatitis requires further investigation.

### Food allergy

Food allergies affect approximately 6% of children under the age of 3 years and 2% of adults, with 1.5 million experiencing peanut allergy in the United States alone [67,68]. The only treatment currently available is strict elimination and avoidance, but unintended ingestion still accounts for 50 to 100 deaths a year in the United States due to peanut products [67]. Another humanized IgG1 monoclonal antibody against IgE, TNX-901, was evaluated in peanut allergy patients. A double-blind, placebo-controlled, randomized trial in 84 patients with proven peanut hypersensitivity evaluated 3 doses of TNX-901 given every 4 weeks for 16 weeks. The mean baseline threshold of sensitivity to peanut flour was 178 to 436 mg for all the groups, which was equivalent to 1/2 to 11/2 peanuts ingested. By the end of treatment, all groups including the placebo group had a greater threshold of peanut tolerability, but only the high-dose TNX-901 group had a significant improvement from a threshold dose of 178 mg (one-half peanut) to 2805 mg (nearly 9 peanuts). However, 25% of the high-dose group had no improvement. There is very little data on the effects of omalizumab on food allergies, and it is not yet approved for this indication. Table 4 summarizes the potential effects of anti-IgE therapy atopic dermatitis and food allergy [29].

**Table 4:** 

Omalizumab and atopic dermatitis
Improved symptoms in some patients
Likely not effective for patients with extremely elevated serum IgE levels
Anti-IgE antibody therapy on food allergy
Only high-dose therapy significantly improved amount of food allergen tolerated
Has not been evaluated with omalizumab

Adapted from Stokes and Casale [29].

### Other

Since becoming widely available, clinicians have exploited omalizumab's immunomodulating properties in the management of several other IgE-mediated disorders as evidenced by case reports. Examples include using the protective effects of omalizumab during venom immunotherapy in a patient who would otherwise be unable to tolerate the immunotherapy [69,70]. Other clinical scenarios in which anti-IgE treatment shows promise include chronic rhinosinusitis,[71] nasal polyposis,[72] chronic urticaria,[73,74] idiopathic angioedema,[75] eosinophil-associated gastrointestinal disorders,[76] mastocytosis,[69,77] latex allergy,[78] and allergic bronchopulmonary aspergillosis [79].

## Dosing

The only FDA-approved anti-IgE therapy is omalizumab (Xolair). The dosing regimen is based on a patient's body weight and total serum IgE level. The recommended dose is 0.016 mg/kg body weight per international unit of IgE every 4 weeks, administered subcutaneously at either 2- or 4-week intervals for adults and adolescents (persons 12 years and older) with moderate-to-severe perennial allergic asthma [80]. For asthma, patients may need a trial of at least 12 weeks before clinical improvement is apparent [81].

Omalizumab is absorbed slowly, reaching peak serum concentrations after an average of 7 to 8 days with no specific uptake of omalizumab by any organ or tissue. In asthma patients, omalizumab serum elimination half-life averaged 26 days. Omalizumab is cleared via IgG as well as elimination of omalizumab/IgE complexes via the liver [80].

Serum total IgE levels (ie, bound and unbound) increase after the first dose due to the formation of omalizumab/IgE complexes, which have a slower elimination rate compared with free IgE. Total IgE levels increased with omalizumab therapy up to 5-fold after 1 month and more than 8 times before omalizumab levels after 3 months of therapy while free IgE levels decreased [82].

The role for eventual dose reduction or cessation of omalizumab remains unclear. A recent study that examined the effects of omalizumab dose reduction and cessation concluded that the marked reduction in serum IgE levels was not maintained at lower doses [83]. In general, after discontinuation of omalizumab dosing, the omalizumab-induced increase in total IgE and decrease in free IgE were reversible. Total IgE levels may take up to a year to achieve pretreatment levels after discontinuation of omalizumab [80].

Beneficial clinical effects, however, may persist for a considerable time despite discontinuing omalizumab. Eighteen patients whose omalizumab was stopped after 6 years of treatment were subsequently followed for 12 to 14 months. Six to 14 months after discontinuing omalizumab, most of the cat- and mite-allergic patients with asthma (13 patients) noted their asthma symptoms to be improved or the same as while on treatment with omalizumab with little or no increase in other asthma medications. Basophil sensitivity to allergen was correspondingly lower (as compared with 15 allergic control subjects) even 12 to 14 months after omalizumab withdrawal as well [84]. These observations may be a reflection of the duration these patients were treated with omalizumab and deserve further investigation.

## Safety

Omalizumab has proven to be a generally well-tolerated medication. The most common adverse event is a local reaction at the injection site that may include burning, pruritis, hives, pain, redness, induration, swelling, warmth, and bruising. In patients with asthma receiving omalizumab or placebo, a local cutaneous reaction was observed in 45% and 43% of subjects, respectively. Severe local cutaneous reactions occurred in 12% of omalizumab-treated subjects and 9% of placebo-treated subjects [80,85]. Other frequent adverse events in patients treated with omalizumab were viral infections (23%), sinusitis (16%), headaches (15%), and pharyngitis (11%). These events occurred at similar rates in omalizumab-treated patients and control patients [80]. Only 6.6% of the total adverse events were felt to be treatment related in the omalizumab group versus 5.6% in the placebo group. Six tenths of a percent of omalizumab-treated patients and 1.1% of placebo-treated patients withdrew from clinical trials due to adverse events.

The safety of omalizumab was evaluated in more than 300 children in a randomized, double-blind, placebo-controlled study [86]. Subjects were treated for 28 weeks followed by a 24-week open label extension. In patients who underwent 52 weeks of treatment with omalizumab, upper respiratory infections and headaches were the most commonly encountered adverse events (47.1% and 42.7%, respectively). During the double-blind 28-week treatment period, the incidence rate of events was similar between omalizumab and placebo. Urticaria was reported in eleven patients (4.9%). With the exception of 1 severe case of urticaria that necessitated withdrawal from the study, all patients with urticaria had either spontaneous remission or resolution with antihistamine treatment. Adverse event incidence during the open label extension was similar to the omalizumab-treated group during the 28-week double-blind phase. The results of this study suggested that omalizumab is well tolerated in children and has a good safety profile.

There were 4127 patients in the original studies who received omalizumab. Of these, 19 (0.46%) developed cancer, whereas 2236 patients received placebo treatment and 5 (0.22%) of this group developed cancer [85]. There were 3726 patients in controlled clinical trials who received omalizumab. Of these, 0.35% developed cancer. None of the differences between these groups were statistically significant. All tumors but 1 (a recurrent non-Hodgkin lymphoma) were solid tumors. Thus, it is very likely that most of these tumors were preexistent. Indeed, a history of cancer was not an exclusion criterion if greater than 3 months before enrollment in the initial studies. Overall, the conclusion of an independent panel of oncologists when they compared the cancer rates for those reported in this population range was that there was no relative risk that was statistically significant for treatment with omalizumab. Nonetheless, postapproval surveillance is appropriate.

In the clinical trials, there was no increase in type 1 hypersensitivity adverse events with an overall incidence of urticaria 1.2% (39/3224) in the omalizumab group and 1.1% (24/2019) in control patients [87]. Clinical trial data documented no evidence of any immune complex disease associated with omalizumab treatment, and in 1723 patients studied, only 1 demonstrated anti-omalizumab antibodies. Postmarketing reports have, however, noted 1 case of serum sickness attributed to omalizumab [88] and 1 case of severe thrombocytopenia. Thrombocytopenia has been added to potential adverse events associated with omalizumab although no causal relationship was established, and routine platelet monitoring is not required. In addition, rare cases of alopecia have been reported (< 0.1%), but no causal relationship to omalizumab has been established [80].

Important to note, rare cases of acute systemic reactions have occurred after omalizumab treatment. These events occurred after the first and multiple doses and usually did not manifest until at least 60 minutes after injection. The mechanisms are unclear [87]. An analysis of anaphylaxis associated with the use of Xolair (omalizumab) was recently published by the Omalizumab Joint Task Force (OJTF). The OJTF is an executive committee formed by the American Academy of Allergy, Asthma and Immunology (AAAAI) and the American College of Allergy, Asthma and Immunology (ACAAI) that reviewed omalizumab clinical trials and postmarketing surveillance data on anaphylaxis and anaphylactoid reactions. The OJTF report included recommendations for physicians who prescribe omalizumab. A summary of the AAAAI/ACAAI OJTF recommendations is included in Table 5. Reviewed data included omalizumab clinical trials and postmarketing reports filed between June 2003 and December 2005, including those filed with the US FDA. The OJTF concluded that during a period when 39,510 patients were receiving omalizumab, there were 41 episodes of anaphylaxis associated with omalizumab administration. This corresponded to an anaphylaxis reporting rate of 0.09% of patients. The OJTF also analyzed the timing of the 41 anaphylactic events. Twenty-two (61%) of these reactions occurred in the first 2 hours after 1 of the first 3 doses. After the fourth dose, most anaphylactic events occurred within 30 minutes of omalizumab administration. On the basis of the timing of these events, the OJTF suggested observation of patients for 2 hours after the first 3 omalizumab treatments and 30 minutes for subsequent injections. Furthermore, administering physicians and other licensed health care providers should have the medications, equipment, and staff to appropriately treat anaphylaxis. Patients taking omalizumab should be trained in the recognition of signs and symptoms of anaphylaxis and in the use of the epinephrine autoinjector [89].

**Table 5:** Summary of AAAAI/ACAAI OJTF Recommendations

Informed consent should be obtained from the patient after discussing the risks, benefits, and alternatives to Xolair (omalizumab).
The patient should be educated regarding the signs, symptoms, and treatment of anaphylaxis.
Patients should be prescribed and educated on the proper use of the epinephrine autoinjector and advised to carry this before Xolair (omalizumab) administration and for the next 24 hours after Xolair (omalizumab) administration.
An assessment of the patient's current health status should be made before each injection to determine whether there were any recent health changes that might require withholding treatment. This assessment should include vital signs and some measure of lung function (eg, peak expiratory flow or FEV_1_).
The OJTF recommends that patients be kept under observation for 30 minutes after each injection. This time should be extended for 2 hours for the first 3 injections based on the data reviewed by the OJTF as well as suggested in the 2007 National Heart, Lung, and Blood Institute Expert Panel Report 3 "Guidelines for the diagnosis and management of asthma." However, this could be modified based on a physician's clinical judgment after discussing risks with the patient.

Adapted from Cox et al [89].

A separate analysis of adverse events reported to the US FDA and to the manufacturers of Xolair (omalizumab) took into account events occurring through December 2006. One hundred twenty-four cases of anaphylaxis were identified and characterized; the estimated number of patients treated during this time was approximately 57, 300. As in the previous analysis, time to onset of anaphylaxis varied widely. One third of cases occurred within 30 minutes of dose administration. Another 1/3 occurred from 30 minutes to 6 hours. The remainder presented more than 6 hours after dose administration. There were also reports of protracted and recurrent episodes. These cases served as the basis for changes in prescribing information including the addition of a Boxed Warning [90].

An additional concern with decreasing serum IgE would be the potential for increased incidence or severity of helminthic infections. A 1-year clinical trial in Brazil was performed with 68 patients treated with omalizumab and 69 placebo controls. The odds ratio for helminth infection was 1.47 (95% confidence interval, 0.74-2.95) indicating that a patient who had infection was anywhere from 0.74 to 2.95 times as likely to have received omalizumab as a patient who did not have an infection. Response to antihelminth therapy was not different between treatment groups. Patients at high risk of helminthic infection should be monitored for infection while on omalizumab therapy [91].

## Conclusions

Selective anti-IgE-humanized monoclonal antibody represents a novel and important therapeutic option for severe asthma and other allergic diseases. The data suggest that omalizumab inhibits activation of mast cells and basophils and decrease the effects of other inflammatory cells such as eosinophils through a variety of mechanisms. This has resulted in clinical improvements in patients with moderate-to-severe allergic asthma, including significant reductions in exacerbations. Omalizumab seems to be a relatively safe and generally well-tolerated medication. In years to come, the role of omalizumab or other anti-IgE antibody strategies in pediatric asthma, nonallergic asthma, food allergy, atopic dermatitis, chronic urticaria with autoantibodies to IgE or the high-affinity IgE receptor, allergic bronchopulmonary aspergillosis, and chronic hyperplastic sinusitis and as an adjuvant to allergen immunotherapy will evolve.

## Notes

Sources of support: none.

## Competing interests

Conflicts of interest: Thomas B. Casale has received support from Genentech and Novartis as an advisory board member, speaker, and investigator.

## References

[B1] Ishizaka K Ishizaka T Terry WD Antigenic structure of gamma-E-globulin and reaginic antibody J Immunol 1967 1 849 858 4169032

[B2] Brownell J Casale TB Anti-IgE therapy Immunol Allergy Clin North Am 2004 1 551 568 v 10.1016/j.iac.2004.06.002 15474858

[B3] Boushey HA Jr Experiences with monoclonal antibody therapy for allergic asthma J Allergy Clin Immunol 2001 1 S77 S83 10.1067/mai.2001.116434 11498677

[B4] Presta LG Lahr SJ Shields RL Humanization of an antibody directed against IgE J Immunol 1993 1 2623 2632 8360482

[B5] Shields RL Whether WR Zioncheck K Inhibition of allergic reactions with antibodies to IgE Int Arch Allergy Immunol 1995 1 308 312 10.1159/000237010 7613156

[B6] Liu J Lester P Builder S Shire SJ Characterization of complex formation by humanized anti-IgE monoclonal antibody and monoclonal human IgE Biochemistry 1995 1 10474 10482 10.1021/bi00033a020 7654701

[B7] Easthope S Jarvis B Omalizumab Drugs 2001 1 253 260 [discussion 61] 10.2165/00003495-200161020-00008 11270941

[B8] MacGlashan D Jr Lichtenstein LM McKenzie-White J Upregulation of FcepsilonRI on human basophils by IgE antibody is mediated by interaction of IgE with FcepsilonRI J Allergy Clin Immunol 1999 1 492 498 10.1016/S0091-6749(99)70399-4 10452777

[B9] Tsicopoulos A Joseph M The role of CD23 in allergic disease Clin Exp Allergy 2000 1 602 605 10.1046/j.1365-2222.2000.00871.x 10792350

[B10] Saini SS MacGlashan DW Jr Sterbinsky SA Down-regulation of human basophil IgE and FC epsilon RI alpha surface densities and mediator release by anti-IgE-infusions is reversible in vitro and in vivo J Immunol 1999 1 5624 5630 10228046

[B11] Lin H Boesel KM Griffith DT Omalizumab rapidly decreases nasal allergic response and FcepsilonRI on basophils J Allergy Clin Immunol 2004 1 297 302 10.1016/j.jaci.2003.11.044 14767445

[B12] Prussin C Griffith DT Boesel KM Lin H Foster B Casale TB Omalizumab treatment downregulates dendritic cell FcepsilonRI expression J Allergy Clin Immunol 2003 1 1147 1154 10.1016/j.jaci.2003.10.003 14657874

[B13] Beck LA Marcotte GV MacGlashan D Togias A Saini S Omalizumabinduced reductions in mast cell Fce psilon RI expression and function J Allergy Clin Immunol 2004 1 527 530 10.1016/j.jaci.2004.06.032 15356552

[B14] Maurer D Fiebiger E Reininger B Fc epsilon receptor I on dendritic cells delivers IgE-bound multivalent antigens into a cathepsin S-dependent pathway of MHC class II presentation J Immunol 1998 1 2731 2739 9743330

[B15] Rissoan MC Soumelis V Kadowaki N Reciprocal control of T helper cell and dendritic cell differentiation Science 1999 1 1183 1186 10.1126/science.283.5405.1183 10024247

[B16] Foster B Metcalfe DD Prussin C Human dendritic cell 1 and dendritic cell 2 subsets express FcepsilonRI: correlation with serum IgE and allergic asthma J Allergy Clin Immunol 2003 1 1132 1138 10.1016/j.jaci.2003.09.011 14657872

[B17] Cheng YX Foster B Holland SM CD2 identifies a monocyte subpopulation with immunoglobulin E-dependent, high-level expression of Fc epsilon RI Clin Exp Allergy 2006 1 1436 1445 10.1111/j.1365-2222.2006.02578.x 17083354 PMC1661841

[B18] Ong YE Menzies-Gow A Barkans J Anti-IgE (omalizumab) inhibits late-phase reactions and inflammatory cells after repeat skin allergen challenge J Allergy Clin Immunol 2005 1 558 564 10.1016/j.jaci.2005.05.035 16159624

[B19] Silkoff PE Romero FA Gupta N Townley RG Milgrom H Exhaled nitric oxide in children with asthma receiving Xolair (omalizumab), a monoclonal anti-immunoglobulin E antibody Pediatrics 2004 1 e308 e312 10.1542/peds.113.4.e308 15060258

[B20] Plewako H Arvidsson M Petruson K The effect of omalizumab on nasal allergic inflammation J Allergy Clin Immunol 2002 1 68 71 10.1067/mai.2002.125488 12110823

[B21] Fahy JV Fleming HE Wong HH The effect of an anti-IgE monoclonal antibody on the early- and late-phase responses to allergen inhalation in asthmatic subjects Am J Respir Crit Care Med 1997 1 1828 1834 10.1164/ajrccm.155.6.9196082 9196082

[B22] Noga O Hanf G Kunkel G Immunological and clinical changes in allergic asthmatics following treatment with omalizumab Int Arch Allergy Immunol 2003 1 46 52 12759489 10.1159/000070434

[B23] Hanf G Brachmann I Kleine-Tebbe J Omalizumab decreased IgE-release and induced changes in cellular immunity in patients with allergic asthma Allergy 2006 1 1141 1144 10.1111/j.1398-9995.2006.01180.x 16918520

[B24] Djukanovic R Wilson SJ Kraft M Effects of treatment with anti-immunoglobulin E antibody omalizumab on airway inflammation in allergic asthma Am J Respir Crit Care Med 2004 1 583 593 10.1164/rccm.200312-1651OC 15172898

[B25] Prieto L Gutierrez V Colas C Effect of omalizumab on adenosine 5Ô-monophosphate responsiveness in subjects with allergic asthma Int Arch Allergy Immunol 2006 1 122 131 10.1159/000090387 16374021

[B26] Boulet LP Chapman KR Cote J Inhibitory effects of an anti-IgE antibody E25 on allergen-induced early asthmatic response Am J Respir Crit Care Med 1997 1 1835 1840 10.1164/ajrccm.155.6.9196083 9196083

[B27] Kitaura J Song J Tsai M Evidence that IgE molecules mediate a spectrum of effects on mast cell survival and activation via aggregation of the FcepsilonRI Proc Natl Acad Sci USA 2003 1 12911 12916 10.1073/pnas.1735525100 14569021 PMC240718

[B28] Noga O Hanf G Brachmann I Effect of omalizumab treatment on peripheral eosinophil and T-lymphocyte function in patients with allergic asthma J Allergy Clin Immunol 2006 1 1493 1499 10.1016/j.jaci.2006.02.028 16751018

[B29] Stokes JR Casale TB Anti-IgE therapy Middleton's allergy principles & practice 2008 7 Philadelphia (PA): Elsevier in press

[B30] Busse W Corren J Lanier BQ Omalizumab, anti-IgE recombinant humanized monoclonal antibody, for the treatment of severe allergic asthma J Allergy Clin Immunol 2001 1 184 190 10.1067/mai.2001.117880 11496232

[B31] Milgrom H Berger W Nayak A Treatment of childhood asthma with anti-immunoglobulin E antibody (omalizumab) Pediatrics 2001 108 E36 10.1542/peds.108.2.e36 11483846

[B32] Soler M Matz J Townley R The anti-IgE antibody omalizumab reduces exacerbations and steroid requirement in allergic asthmatics Eur Respir J 2001 1 254 261 10.1183/09031936.01.00092101 11529281

[B33] Corren J Casale T Deniz Y Ashby M Omalizumab, a recombinant humanized anti-IgE antibody, reduces asthma-related emergency room visits and hospitalizations in patients with allergic asthma J Allergy Clin Immunol 2003 1 87 90 10.1067/mai.2003.49 12532101

[B34] Bousquet J Wenzel S Holgate S Freeman P Fox H Predicting response to omalizumab, an anti-IgE antibody, in patients with allergic asthma Chest 2004 1 1378 1386 10.1378/chest.125.4.1378 15078749

[B35] Holgate S Bousquet J Wenzel S Fox H Liu J Castellsague J Efficacy of omalizumab, an anti-immunoglobulin E antibody, in patients with allergic asthma at high risk of serious asthma-related morbidity and mortality Curr Med Res Opin 2001 1 233 240 11922396

[B36] Holgate ST Chuchalin AG Hebert J Efficacy and safety of a recombinant anti-immunoglobulin E antibody (omalizumab) in severe allergic asthma Clin Exp Allergy 2004 1 632 638 10.1111/j.1365-2222.2004.1916.x 15080818

[B37] Oba Y Salzman GA Cost-effectiveness analysis of omalizumab in adults and adolescents with moderate-to-severe allergic asthma J Allergy Clin Immunol 2004 1 265 269 10.1016/j.jaci.2004.05.049 15316501

[B38] Ayres JG Higgins B Chilvers ER Ayre G Blogg M Fox H Efficacy and tolerability of anti-immunoglobulin E therapy with omalizumab in patients with poorly controlled (moderate-to-severe) allergic asthma Allergy 2004 1 701 708 10.1111/j.1398-9995.2004.00533.x 15180756

[B39] Humbert M Beasley R Ayres J Benefits of omalizumab as add-on therapy in patients with severe persistent asthma who are inadequately controlled despite best available therapy (GINA 2002 step 4 treatment): INNOVATE Allergy 2005 1 309 316 10.1111/j.1398-9995.2004.00772.x 15679715

[B40] Bousquet J Cabrera P Berkman N The effect of treatment with omalizumab, an anti-IgE antibody, on asthma exacerbations and emergency medical visits in patients with severe persistent asthma Allergy 2005 1 302 308 10.1111/j.1398-9995.2004.00770.x 15679714

[B41] Casale TB Condemi J LaForce C Effect of omalizumab on symptoms of seasonal allergic rhinitis: a randomized controlled trial JAMA 2001 1 2956 2967 10.1001/jama.286.23.2956 11743836

[B42] Adelroth E Rak S Haahtela T Recombinant humanized mAb-E25, an anti-IgE mAb, in birch pollen-induced seasonal allergic rhinitis J Allergy Clin Immunol 2000 1 253 259 10.1067/mai.2000.108310 10932067

[B43] Hanf G Noga O O'Connor A Kunkel G Omalizumab inhibits allergen challenge-induced nasal response Eur Respir J 2004 1 414 418 10.1183/09031936.04.00024504 15065831

[B44] Okubo K Ogino S Nagakura T Ishikawa T Omalizumab is effective and safe in the treatment of Japanese cedar pollen-induced seasonal allergic rhinitis Allergol Int 2006 1 379 386 10.2332/allergolint.55.379 17130680

[B45] Nagakura T Ogino S Okubo K Sato N Takahashi M Ishikawa T Omalizumab is more effective than suplatast tosilate in the treatment of Japanese cedar pollen-induced seasonal allergic rhinitis Clin Exp Allergy 2008 1 329 337 18070163 10.1111/j.1365-2222.2007.02894.x

[B46] Chervinsky P Casale T Townley R Omalizumab, an anti-IgE antibody, in the treatment of adults and adolescents with perennial allergic rhinitis Ann Allergy Asthma Immunol 2003 1 160 167 10.1016/S1081-1206(10)62171-0 12952110

[B47] Bousquet J Van Cauwenberge P Khaltaev N Allergic rhinitis and its impact on asthma J Allergy Clin Immunol 2001 1 S147 334 10.1067/mai.2001.118891 11707753

[B48] Corren J Manning BE Thompson SF Hennessy S Strom BL Rhinitis therapy and the prevention of hospital care for asthma: a case-control study J Allergy Clin Immunol 2004 1 415 419 10.1016/j.jaci.2003.11.034 15007339

[B49] Dykewicz M Rhinitis and sinusitis.Implications for severe asthma Immunol Allergy Clin North Am 2001 1 427 436 10.1016/S0889-8561(05)70219-8

[B50] Greenberger PA Interactions between rhinitis and asthma Allergy Asthma Proc 2004 1 89 93 15176491

[B51] Vignola AM Humbert M Bousquet J Efficacy and tolerability of anti-immunoglobulin E therapy with omalizumab in patients with concomitant allergic asthma and persistent allergic rhinitis: SOLAR Allergy 2004 1 709 717 10.1111/j.1398-9995.2004.00550.x 15180757

[B52] Akdis CA Blesken T Akdis M Wuthrich B Blaser K Role of interleukin 10 in specific immunotherapy J Clin Invest 1998 1 98 106 10.1172/JCI2250 9649562 PMC509070

[B53] Muller U Akdis CA Fricker M Successful immunotherapy with T-cell epitope peptides of bee venom phospholipase A2 induces specific T-cell anergy in patients allergic to bee venom J Allergy Clin Immunol 1998 1 747 754 10.1016/S0091-6749(98)70402-6 9648701

[B54] Yssel H Lecart S Pene J Regulatory T cells and allergic asthma Microbes Infect 2001 1 899 904 10.1016/S1286-4579(01)01450-2 11564437

[B55] Guerra F Carracedo J Solana-Lara R Sanchez-Guijo P Ramirez R Th2 lymphocytes from atopic patients treated with immunotherapy undergo rapid apoptosis after culture with specific allergens J Allergy Clin Immunol 2001 1 647 653 10.1067/mai.2001.112263 11295653

[B56] Gehlhar K Schlaak M Becker W Bufe A Monitoring allergen immunotherapy of pollen-allergic patients: the ratio of allergen-specific IgG4 to IgG1 correlates with clinical outcome Clin Exp Allergy 1999 1 497 506 10.1046/j.1365-2222.1999.00525.x 10202364

[B57] Durham SR Till SJ Immunologic changes associated with allergen immunotherapy J Allergy Clin Immunol 1998 1 157 164 10.1016/S0091-6749(98)70079-X 9723654

[B58] Kuehr J Brauburger J Zielen S Efficacy of combination treatment with anti-IgE plus specific immunotherapy in polysensitized children and adolescents with seasonal allergic rhinitis J Allergy Clin Immunol 2002 1 274 280 10.1067/mai.2002.121949 11842297

[B59] Rolinck-Werninghaus C Hamelmann E Keil T The co-seasonal application of anti-IgE after preseasonal specific immunotherapy decreases ocular and nasal symptom scores and rescue medication use in grass pollen allergic children Allergy 2004 1 973 979 10.1111/j.1398-9995.2004.00552.x 15291906

[B60] Casale TB Busse WW Kline JN Omalizumab pretreatment decreases acute reactions after rush immunotherapy for ragweed-induced seasonal allergic rhinitis J Allergy Clin Immunol 2006 1 134 140 10.1016/j.jaci.2005.09.036 16387596

[B61] Leung DY Nicklas RA Li JT Disease management of atopic dermatitis: an updated practice parameter. Joint Task Force on Practice Parameters Ann Allergy Asthma Immunol 2004 1 S1 21 15478395 10.1016/s1081-1206(10)61385-3

[B62] Novak N Bieber T Allergic and nonallergic forms of atopic diseases J Allergy Clin Immunol 2003 1 252 262 10.1067/mai.2003.1595 12897728

[B63] Lane JE Cheyney JM Lane TN Kent DE Cohen DJ Treatment of recalcitrant atopic dermatitis with omalizumab J Am Acad Dermatol 2006 1 68 72 10.1016/j.jaad.2005.09.030 16384758

[B64] Vigo PG Girgis KR Pfuetze BL Critchlow ME Fisher J Hussain I Efficacy of anti-IgE therapy in patients with atopic dermatitis J Am Acad Dermatol 2006 1 168 170 10.1016/j.jaad.2005.12.045 16781320

[B65] Krathen RA Hsu S Failure of omalizumab for treatment of severe adult atopic dermatitis J Am Acad Dermatol 2005 1 338 340 10.1016/j.jaad.2005.02.014 16021135

[B66] Belloni B Ziai M Lim A Low-dose anti-IgE therapy in patients with atopic eczema with high serum IgE levels J Allergy Clin Immunol 2007 1 1223 1225 10.1016/j.jaci.2007.08.060 17936892

[B67] Leung DY Sampson HA Yunginger JW Effect of anti-IgE therapy in patients with peanut allergy N Engl J Med 2003 1 986 993 10.1056/NEJMoa022613 12637608

[B68] Sampson HA Food allergy J Allergy Clin Immunol 2003 1 S540 S547 10.1067/mai.2003.134 12592300

[B69] Kontou-Fili K High omalizumab dose controls recurrent reactions to venom immunotherapy in indolent systemic mastocytosis Allergy 2008 1 376 378 10.1111/j.1398-9995.2007.01604.x 18269681

[B70] Schulze J Rose M Zielen S Beekeepers anaphylaxis: successful immunotherapy covered by omalizumab Allergy 2007 1 963 964 10.1111/j.1398-9995.2007.01388.x 17484729

[B71] Grundmann SA Hemfort PB Luger TA Brehler R Anti-IgE (omalizumab): a new therapeutic approach for chronic rhinosinusitis J Allergy Clin Immunol 2008 1 257 258 10.1016/j.jaci.2007.09.036 18206513

[B72] Penn R Mikula S The role of anti-IgE immunoglobulin therapy in nasal polyposis: a pilot study Am J Rhinol 2007 1 428 432 10.2500/ajr.2007.21.3060 17882911

[B73] Metz M Bergmann P Zuberbier T Maurer M Successful treatment of cholinergic urticaria with anti-immunoglobulin E therapy Allergy 2008 1 247 249 10.1111/j.1398-9995.2007.01591.x 18186820

[B74] Spector SL Tan RA Effect of omalizumab on patients with chronic urticaria Ann Allergy Asthma Immunol 2007 1 190 193 10.1016/S1081-1206(10)60644-8 17718108

[B75] Sands MF Blume JW Schwartz SA Successful treatment of 3 patients with recurrent idiopathic angioedema with omalizumab J Allergy Clin Immunol 2007 1 979 981 10.1016/j.jaci.2007.07.041 17931567

[B76] Foroughi S Foster B Kim N Anti-IgE treatment of eosinophilassociated gastrointestinal disorders J Allergy Clin Immunol 2007 1 594 601 10.1016/j.jaci.2007.06.015 17765756 PMC2768344

[B77] Siebenhaar F Kuhn W Zuberbier T Maurer M Successful treatment of cutaneous mastocytosis and Meniere disease with anti-IgE therapy J Allergy Clin Immunol 2007 1 213 215 10.1016/j.jaci.2007.05.011 17544095

[B78] Leynadier F Doudou O Gaouar H Effect of omalizumab in health care workers with occupational latex allergy J Allergy Clin Immunol 2004 1 360 361 10.1016/j.jaci.2003.11.020 14767458

[B79] van der Ent CK Hoekstra H Rijkers GT Successful treatment of allergic bronchopulmonary aspergillosis with recombinant anti-IgE antibody Thorax 2007 1 276 277 10.1136/thx.2004.035519 17329558 PMC2117163

[B80] Xolair (omalizumab) 2006 East Hanover (NJ): Aventis [package insert]

[B81] Strunk RC Bloomberg GR Omalizumab for asthma N Engl J Med 2006 1 2689 2695 10.1056/NEJMct055184 16790701

[B82] Hamilton RG Accuracy of US Food and Drug Administration-cleared IgE antibody assays in the presence of anti-IgE (omalizumab) J Allergy Clin Immunol 2006 1 759 766 10.1016/j.jaci.2006.01.012 16630931

[B83] Corren J Shapiro G Reimann J Allergen skin tests and free IgE levels during reduction and cessation of omalizumab therapy J Allergy Clin Immunol 2008 1 506 511 10.1016/j.jaci.2007.11.026 18269927

[B84] Nopp A Johansson SG Ankerst J Palmqvist M Oman H CD-sens and clinical changes during withdrawal of Xolair after 6 years of treatment Allergy 2007 1 1175 1181 10.1111/j.1398-9995.2007.01476.x 17845588

[B85] Prescribing information: summary of product characteristics Available at: http://www.xolair.com

[B86] Berger W Gupta N McAlary M Fowler-Taylor A Evaluation of long-term safety of the anti-IgE antibody, omalizumab, in children with allergic asthma Ann Allergy Asthma Immunol 2003 1 182 188 10.1016/S1081-1206(10)62175-8 12952113

[B87] Chipps B Systemic reaction to omalizumab Ann Allergy Asthma Immunol 2006 1 267 10.1016/S1081-1206(10)60027-0 16937765

[B88] Pilette C Coppens N Houssiau FA Rodenstein DO Severe serum sickness-like syndrome after omalizumab therapy for asthma J Allergy Clin Immunol 2007 1 972 973 10.1016/j.jaci.2007.06.038 17716723

[B89] Cox L Platts-Mills TA Finegold I Schwartz LB Simons FE Wallace DV American Academy of Allergy, Asthma & Immunology/American College of Allergy, Asthma and Immunology Joint Task Force Report on omalizumab-associated anaphylaxis J Allergy Clin Immunol 2007 1 1373 1377 10.1016/j.jaci.2007.09.032 17996286

[B90] Limb SL Starke PR Lee CE Chowdhury BA Delayed onset and protracted progression of anaphylaxis after omalizumab administration in patients with asthma J Allergy Clin Immunol 2007 1 1378 1381 10.1016/j.jaci.2007.09.022 17936893

[B91] Cruz AA Lima F Sarinho E Safety of anti-immunoglobulin E therapy with omalizumab in allergic patients at risk of geohelminth infection Clin Exp Allergy 2007 1 197 207 10.1111/j.1365-2222.2007.02650.x 17250692 PMC1859973

